# No Easy Talk: A Mixed Methods Study of Doctor Reported Barriers to Conducting Effective End-of-Life Conversations with Diverse Patients

**DOI:** 10.1371/journal.pone.0122321

**Published:** 2015-04-22

**Authors:** Vyjeyanthi S. Periyakoil, Eric Neri, Helena Kraemer

**Affiliations:** 1 Stanford University School of Medicine, Palo Alto, CA, 94304, United States of America; 2 VA Palo Alto Health Care System, Palo Alto, CA, 94304, United States of America; Supportive care, Early DIagnosis and Advanced disease (SEDA) research group, UNITED KINGDOM

## Abstract

**Objective:**

Though most patients wish to discuss end-of-life (EOL) issues, doctors are reluctant to conduct end-of-life conversations. Little is known about the barriers doctors face in conducting effective EOL conversations with diverse patients. This mixed methods study was undertaken to empirically identify barriers faced by doctors (if any) in conducting effective EOL conversations with diverse patients and to determine if the doctors’ age, gender, ethnicity and medical sub-specialty influenced the barriers reported.

**Design:**

Mixed-methods study of multi-specialty doctors caring for diverse, seriously ill patients in two large academic medical centers at the end of the training; data were collected from 2010 to 2012.

**Outcomes:**

Doctor-reported barriers to EOL conversations with diverse patients.

**Results:**

1040 of 1234 potential subjects (84.3%) participated. 29 participants were designated as the development cohort for coding and grounded theory analyses to identify primary barriers. The codes were validated by analyses of responses from 50 randomly drawn subjects from the validation cohort (n= 996 doctors). Qualitative responses from the validation cohort were coded and analyzed using quantitative methods. Only 0.01 % doctors reported no barriers to conducting EOL conversations with patients. 99.99% doctors reported barriers with 85.7% finding it very challenging to conduct EOL conversations with all patients and especially so with patients whose ethnicity was different than their own. Asian-American doctors reported the most struggles (91.3%), followed by African Americans (85.3%), Caucasians (83.5%) and Hispanic Americans (79.3%) in conducting EOL conversations with their patients. The biggest doctor-reported barriers to effective EOL conversations are (i) language and medical interpretation issues, (ii) patient/family religio-spiritual beliefs about death and dying, (iii) doctors’ ignorance of patients’ cultural beliefs, values and practices, (iv) patient/family's cultural differences in truth handling and decision making, (v) patients’ limited health literacy and (vi) patients’ mistrust of doctors and the health care system. The doctors' ethnicity (Chi-Square = 12.77, DF = 4, p = 0.0125) and medical subspecialty (Chi-Square = 19.33, DF = 10, p =0.036) influenced their reported barriers. Friedman’s test used to examine participants relative ranking of the barriers across sub-groups identified significant differences by age group (F statistic = 303.5, DF = 5, p < 0.0001) and medical sub-specialty (F statistic =163.7, DF = 5, p < 0.0001).

**Conclusions and Relevance:**

Doctors report struggles with conducting effective EOL conversations with all patients and especially with those whose ethnicity is different from their own. It is vital to identify strategies to mitigate barriers doctors encounter in conducting effective EOL conversations with seriously ill patients and their families.

## Introduction

By 2030, there will be 71 million older Americans accounting for roughly 20% of the U.S. population [1]. While biomedical advances have resulted in an unprecedented growth in the number and proportion of older adults, most (an estimated 80%) bear the burden of chronic illness(es) for several years before eventually succumbing to it. Costs of caring for patients with chronic illnesses have escalated the U.S. health care costs which were 17.7% of the Gross Domestic Product in 2011 [2] (250% more than the average health care spending of most developed nations). These costs are projected to further increase by an alarming 25% in the next two decades due to the demographic shifts in a greying nation. A disproportionate amount of the total Medicare budget [3] is expended on patients in the last two years of their life on repeated hospitalizations. An estimated 78% of costs [4] in the final year of life is spent in the very last month of life on ineffective and burdensome high-intensity treatments which are associated with poor quality of death [5]. Better health outcomes do not necessarily accompany these higher spending patterns [3] leading to the inference that judicious cost curbing is possible without compromising the quality of care at the EOL.

In analyzing the growing population patterns, it is clear that the minority populations are growing rapidly, and the U.S. is projected [6,7] to become a minority-majority nation by 2043. The ethnogeriatric population is exploding into a ‘*silver-brown tsunami*’ of older Americans who are living longer while enduring the burdens of chronic illnesses for many years. Data show that ethnic patients are more likely to consume ineffective and burdensome high-intensity treatments at the EOL, incur higher end-of-life costs[8] and are less likely[9–11] to utilize hospice care. EOL decision making for ethnic patients is impaired by poor communication and lack of understanding about the treatment options[11–13]. Compared to Whites, African-American decedents report more problems with physician communication[11].

Doctors do not necessarily believe that high-intensity treatments are desirable at the EOL. In our previous work[14] we have demonstrated that most doctors personally prefer to forego high-intensity treatments and wish to die gently. One key reason for why terminally ill patients are subjected to ineffective and burdensome treatments is the lack of effective EOL conversations that elicit the patient’s values and preferences early in the chronic illness trajectory and what matters most to them. Research[5] shows that patients who have EOL conversations with their physicians are less likely to experience physical distress at life’s end, less likely to undergo high-intensity interventions, less likely to die in the Intensive Care Unit, more likely to receive outpatient hospice care and be referred to hospice earlier.

EOL conversations *per se* are very sensitive communication encounters and conducting these may be very challenging[15–17]. To our knowledge, doctor-reported barriers (if any) to conducting effective EOL conversations have not been well studied. This large mixed-methods[18] study was conducted to determine if doctors struggle with conducting EOL conversations with their patients and to better characterize barriers doctors may face in conducting such conversations with diverse patients.

## Methods

### Study sample

Multi-specialty doctors who care for seriously ill patients in two large training hospitals in California (Stanford Hospital and Clinics and the VA Palo Alto) participated at the end of the clinical training year just before graduation in the academic years of 2010 and 2011 (data were collected from calendar years 2010 through 2012). We specifically did purposive sampling of trainees from various sub-specialties just before graduation as these doctors have very high practice volumes and care for numerous seriously ill persons from various ethnic backgrounds. Of the 1234 eligible participants, 1040 participated (84.3% response rate).

### Data collection

The questionnaire was administered one time online and no personal health identifiers were collected in an effort to promote participant confidentiality and honest responses without concerns about individual scrutiny. There was no repeated contact of participants. The Stanford Institutional Review Board approval was obtained to analyze the data presented in this paper.

#### Questions and prompts used in data collection

- Have you encountered any barriers to conducting effective EOL conversations with seriously ill patients and families?
- If yes, to what extent conducting effective EOL conversations with patients and families who belong to a different cultural/ethnic background was challenging (Likert response choices: not at all, somewhat, quite a bit and a great deal).
- Please list the top three barriers (if any) that you have faced in conducting effective EOL conversations including those with patients and families who belong to an ethnic/racial group different from your own.

The participants’ age, gender, ethnicity and medical sub-specialty information and its influence on their perceptions were analyzed, all with a view of improving the training and resources of doctors to deal with EOL problems.

### Data analysis

#### Qualitative data analyses of development cohort to identify key codes

**Fig 1:**
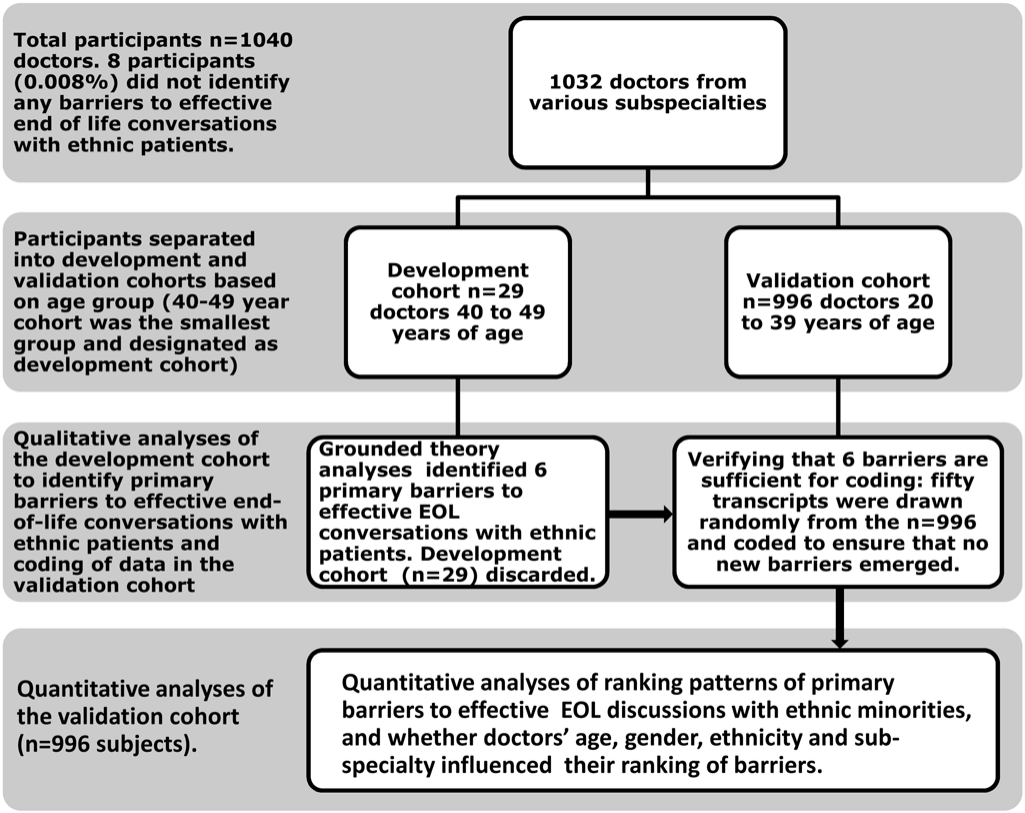
STUDY FLOW: The study subjects were divided into code development and validation cohorts. After identifying the 6 primary barriers using the development cohort, these participants were not used in subsequent analyses. The flow of study participants is shown in the figure.

The data of the participants who reported any barriers to effective EOL conversations was sorted based on age group (20–29 years, 30–39 years. and 40 years or older). 29 participants were 40 to 49 years old, and this cohort was designated to be the development cohort (see Fig 1) for purposes of qualitative analyses and code development. The open ended responses of the 29 development cohort participants were transcribed, coded and analyzed using Microsoft Access and NVivo 7 (QSR International Pty Ltd., Melbourne Australia). Grounded theory techniques[19–20] of intense open and axial coding were used to identify key barriers to conducting effective EOL conversations with ethnic minority patients. First, using an open coding approach, one of the authors (VSP) and a trained research assistant independently coded the transcripts of the development cohort. During open coding, recurrent barriers were identified and characterized. During axial coding, the barrier categories were further defined, and the relationships between them were characterized. Next, the coders compared their assignment of codes and a third investigator (HCK) mediated any discrepancies between codes assigned until agreement was reached. Efforts to maintain the validity of the qualitative data included the following: to evaluate inter-coder reliability, the exact assignment of codes was assessed for 100% of the development cohort transcripts; agreement was found to be greater than 90%, which was deemed to be comparable with previous work. After 19 transcripts had been coded, no new codes emerged from the next five transcripts (data saturation had been reached). To confirm data saturation, the last five transcripts in the development cohort were coded, with no new code labels emerging. At the end of this process, 6 primary barriers were identified and the development cohort was discarded and not used in subsequent analyses. Actual words and phrases that participants used were used to name the barriers as feasible (see Table 1 for barriers with exemplars).

**Table 1:** Top six barriers (with exemplars) to effective end-of-life conversations with ethnic minorities according to multi-ethnic, multi-specialty doctors.

**Barrier 1: Language and medical interpretation issues**
“Language barrier: inability to communicate with patients/families and ensure they understand the discussion”
“Ability to communicate the message in the different language, nuances about meaning of certain words that may not be well accepted in the (other) culture”
“Inherent language barrier—***medical-ese*** is difficult enough for fluent speakers”
“(It is) hard to talk about sensitive topics through an interpreter”
“Difficulty in translation, sometimes interpreters may not exactly translate the feeling and meaning of a conversation.”
“Connecting emotionally to the patient and/or family through an interpreter (is a barrier)”
“Communicating end-of-life discussions through a translator is extremely awkward.”
**Barrier 2: Patient/ family religious and spiritual beliefs about death and dying**
“Religious cultural values that may lead people to prefer life-sustaining treatments that we may see as futile.”
“Reconciling religious "obstructions" to a Do Not Resuscitate status.”
“Hoping for religious miracle.”
“Discussions of God and afterlife beliefs”
**Barrier 3: Doctors’ ignorance of patients’ cultural beliefs, values and practices**
“Doctors not understanding the cultural values surrounding end-of-life care for a patient with a different ethnic/religious background.”
“Unfamiliar with social norms for showing sympathy, hug? cry?”
“Cultural norms that differ from my own causing me to inadvertently offend the patient or his/her family.”
“Not understanding which topics might be taboo.”
“Not knowing how to discuss goals in a way that makes sense to someone with different views about death based on different beliefs about spirituality and afterlife.”
**Barrier 4: Cultural differences in truth handling and decision making**
“Certain groups feel that honesty and frankness is not good for the emotional outlook on life for the patient and they do not want the physician to be frank with the patient in discussing end-of-life issues at all.”
“I think it's fair to say that some cultures approach (conversations about) death as something to be avoided at all costs, which is not necessarily how i, as a health care provider, feel about it.”
“In some cultures (i.e. Asians), patients may not want their diagnoses/prognoses discussed with them directly and will instead appoint a family member as surrogate decision maker. It can become difficult however, to be sure that that family member is acting in the best interest of the patient and acting with the patient's preferences in mind vs. their own.”
“Different opinions on the role of patient autonomy in making end-of-life decisions understanding that in some cultures the decision making may fall to a different member of the family than the patient.”
“Eliciting the personal wishes of a female from a culture in which men make all the decisions can be difficult.”
**Barrier 5: Patient/family's limited health literacy**
“Certain medical terms may be difficult to explain in a way the patient can understand.”
“They may not be used to the health system they find themselves in and it may be overlooked that they lack what we would consider common knowledge”
“Incomplete understanding of what resources/therapies that can be versus should be provided for a patient.”
“Misunderstanding what is described by resuscitation, thinking it means we are giving up completely on treatment”
**Barrier 6: Patient/family’s mistrust of doctors and the health care system**
“Mistrust/misunderstanding of the motivations of the medical community”
“Some groups feel more marginalized in the community at large and this makes them more distrustful of the medical system as a whole.”
“Patients may believe that care is being "withdrawn" from their loved one because of racism.”
“Certain cultures lack trust in the medical profession, do not believe physicians have their best interests at heart.”
“Fears of abandonment or self-interested medical professionals”.

#### Code validation

The validation cohort consisted of 996 participants age range of from 20–39 years (see Table 2 for demographics). Of the 996 transcripts, 50 were drawn randomly and coded independently by the coders (VSP and RS) for the presence or absence of each of the six primary barriers identified, as well for any new codes. The two coders then met and compared codes for each transcript and determined that no new recurrent codes emerged and that the six primary barriers identified earlier were sufficient to appropriately code the entire validation dataset. (See Fig 1 for study flow.)

**Table 2:** Demographic characteristics showing validation cohort participant gender, age, race, ethnicity and subspecialty (n = 996).

Category		N	%
**Gender**			
	Female	501	50.3
	Male	495	49.7
**Age**			
	20–29 years	444	44.6
	30–39 years	552	55.4
**Race/Ethnicity**			
	Caucasian	485	48.7
	Latino-American	58	5.8
	African-American	34	3.4
	Asian	335	33.6
	Mixed race and ethnicity	84	8.4
**Medical Subspecialty**			
	Anesthesiology	95	9.5
	Emergency Medicine	29	2.9
	Internal Medicine	289	29.0
	Neurology	32	3.2
	Obstetrics & Gynecology	25	2.5
	Physical Medicine and Rehabilitation	22	2.2
	Pathology	50	5.0
	Pediatrics	140	14.1
	Psychiatry	52	5.2
	Surgery	188	18.9
	Radiation & Nuclear Medicine	74	7.4

#### Qualitative coding of the validation cohort (n = 996 subjects)

Next, the validation dataset responses were coded independently by the two coders qualitatively for the presence or absence of the six key primary barriers to conducting effective end-of-life conversations identified by earlier qualitative analyses using grounded theory methods. Upon completion the codes were compared for inter-rater reliability. The agreement was 94%, which was deemed to be at par with previous work[21]. All discrepancies in codes were reviewed by both coders with one of the authors (HCK) and discussed until consensus was reached.

#### Quantitative analyses of validation cohort responses

The primary six barriers were rank ordered according to the responses of the each of the 996 cohort subjects (with ties, particularly for those barriers not mentioned). The data were imported into SAS (SAS 9.3, SAS Inc., North Carolina) for quantitative analyses. The participants’ Likert response to the statement “conducting effective EOL conversations with patients and families who belong to a different cultural/ethnic background is challenging” was analyzed using the Mann-Whitney-Wilcoxon[22,23] test (subgroup analysis by gender and age group) and the Kruskal-Wallis test[24] (subgroup analyses by ethnicity/race and medical sub-specialty). The Friedman test[25] was used to compare subgroups (age, gender, ethnicity and sub-specialty) and determine if any of the six primary barriers was ranked consistently higher or lower.

## Results

Of the total 1040 participants, only 8 doctors (<0.1%) did not report any barriers to conducting effective EOL conversations. 1032 (99.9%) doctors reported barriers to conducting effective EOL conversations and most doctors (85.7%) stated that conducting effective EOL conversations with ethnic patients to be “a great deal” or “quite a bit” challenging. Subgroup analyses showed no significant differences by gender or age but revealed significant differences by ethnicity (Chi-Square = 12.8, DF = 4, p = 0.01) and by medical subspecialty (Chi-Square = 19.3, DF = 10, p = 0.04). Asian doctors reported the most struggles (91.3%), followed by African American doctors (85.3%), Caucasian doctors (83.5%) and finally Hispanic Latino doctors (79.3%).

**Fig 2:**
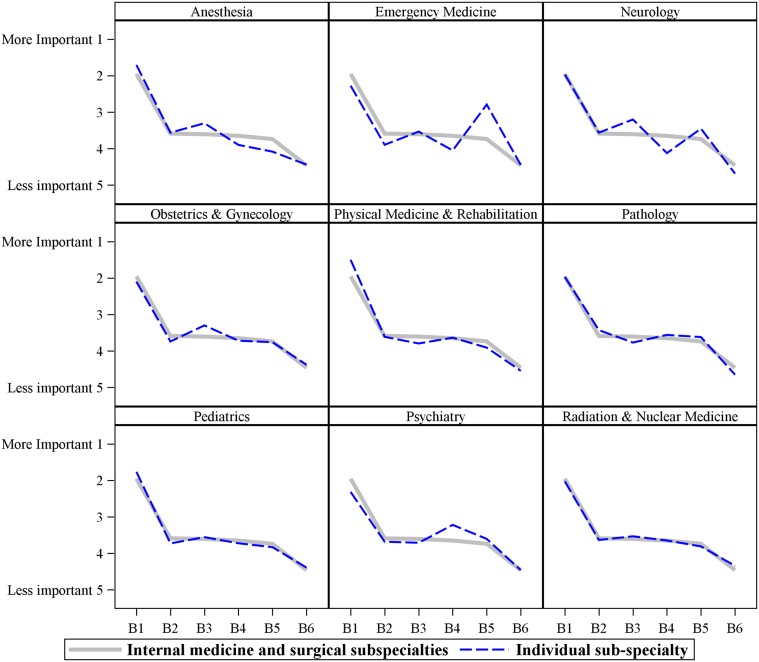
Graphic shows how doctors in various medical subspecialties rate the relative importance of the 6 primary barriers to effective EOL conversations with ethnic patients. The dotted line represents the individual sub-specialty. The solid line represents the average ranks for doctors from the two largest sub-specialties, namely Internal Medicine and Surgery. The biggest differences across sub-specialties were seen in Emergency Medicine, Neurology, Psychiatry and Anesthesia and these are shown in comparison with the two largest subspecialties (Medicine and Surgery) as line graphs. B1 to B6 represent the top six barriers to effective EOL conversations with ethnic patients. B1 = Language and medical interpretation issues; B2 = Patient/ family religious and spiritual beliefs about death and dying; B3 = Doctors’ ignorance of patients’ cultural beliefs, values and practices; B4 = Cultural differences in truth handling and decision making; B5 = Patient/family's limited health literacy; and B6 = Patient/family’s mistrust of doctors and the health care system.

Next, the Friedman’s[25, 26] test was used to examine the differences in rankings for the 6 primary barriers across sub-groups of participants. There were no significant differences by gender or ethnicity. There were significant differences in barrier ranking by age group (F statistic = 303.5, DF = 5, p < 0.0001). Younger doctors felt that patient/family’s limited health literacy was a bigger barrier compared to the older doctors in our sample. There were also significant differences based on medical sub-specialty (F statistic = 163.7, DF = 5, p < 0.0001). (See Fig 2). The largest sub-specialty groups in the validation cohort were Internal Medicine (n = 289) and Surgery (n = 188). Of the six primary barriers, all sub-specialties identified "language and medical interpretation issues" to be the most problematic and “patient/family’s mistrust of doctors and the health care system” to be the least problematic of the barriers to effective EOL conversations with diverse patients. The biggest differences across sub-specialties were seen in Emergency Medicine, Neurology, Psychiatry and Anesthesia. Emergency Medicine doctors rated the patient/family’s limited health literacy barrier as being more problematic compared to doctors in general. Neurologists rated doctors’ ignorance of patients’ cultural beliefs, values and practices as a more problematic and cultural differences in truth handling and decision making as less problematic as compared to doctors in general. Psychiatrists stated that cultural differences in truth handling and decision-making were more problematic compared to doctors in general. Anesthesiologists felt that patient/family's limited health literacy was less of a barrier compared to doctors in general. To note, 26 of the 29 (89%) of the Emergency Medicine doctors in our cohort were 20–29 years old and it is possible that the differences noted were a function of age in addition to sub-specialty.

## Discussion

Almost all the doctors in our study reported barriers to conducting effective EOL conversations with patients and 85.7% stated that conducting EOL discussions with patients was very challenging.

### Barrier 1: Language and medical interpretation issues

Medical jargon is difficult to translate into other languages (as equivalent words may not exist) and approximate translations do not convey the true meaning and may lead to misunderstandings and miscommunication. Doctors felt that the technical medical terms used in EOL discussions (cardio-pulmonary resuscitation, ventilators) or vague phrases like "heroic measures" made it very challenging to have these discussions with persons of Limited English Proficiency (LEP). The doctors in our study identified medical interpreters (MI) services as a barrier to effective EOL conversations for the following reasons: (i) logistical difficulties encountered with in-person/phone/video MIs, (ii) conversations involving MIs were time intensive, (iii) using MIs as communication intermediaries psychologically distanced the doctors from their patients, (iv) the variability in quality and accuracy of the MIs, (v) sometimes MIs interfered with the diplomacy of the doctor-patient encounter by giving advice to the patient or by assuming the role of a patient advocate.

As the US population is becoming more diverse, it is imperative that we train doctors and medical interpreters to work together respectfully and effectively following standard guidelines[27–28]. Furthermore, system triggers need to be built to automatically identify patients with LEP and schedule adequate clinical encounter time for doctors to work with MIs and these patients. Additionally system changes need to be made so that all doctors will be able to use special billing codes like the interactive complexity add-on Current Procedural Terminology[29] codes when working with MIs and be adequately reimbursed for these time-intensive and complex encounters.

### Barrier 2: Patient/ family religious and spiritual beliefs about death and dying

Doctors reported that religious and spiritual beliefs greatly influenced how ethnic patients perceived EOL issues ranging from (i) an unwillingness to discuss EOL issues or plan ahead due to their beliefs that the timing and nature of a person's death should be determined “by the will of God” and humans should not tamper with the process (ii) religious taboos about withholding/withdrawing high intensity interventions at the EOL (iii) as families were praying for miracles and thus refused to engage in EOL discussions and planning even in the face of impending death; and (iv) patient/family beliefs that the specific circumstances around the death impacts the patient’s afterlife and they were reluctant to participate in actions that may in some way alter the timing and course of death. Doctors need to be trained to work effectively with patients and families from diverse religio-spiritual backgrounds and to consult and partner with chaplains and community spiritual leaders in providing necessary support to seriously ill patients and families to facilitate quality EOL decisions.

### Barrier 3: Doctors’ ignorance of patients’ cultural beliefs, values and practices

The doctors felt that their ignorance about the diverse cultural values around death and dying was the third biggest barrier to effective EOL conversations with seriously ill patients and their families from diverse cultures. This lack of knowledge led to the doctors' (i) inability to empathize with the ethnic patients' cultural values that influence EOL decisions, and (ii) committing cultural *faux pas* by discussing taboo topics which inadvertently offended the patient/family and undermined the therapeutic relationship. Doctors need to be trained[15–17] on how to effectively conduct EOL discussions in a culturally sensitive and competent manner with seriously ill persons from various cultural and ethnic backgrounds. Such training could be incorporated as a longitudinal curricular thread across the continuum of medical education ranging from undergraduate to postgraduate training and as an essential component of continuing medical education.

### Barrier 4: Cultural differences in truth handling and decision making

Doctors stated that in some cultures, the patient/family believed that even speaking about death and dying would invoke death sooner and therefore refused to engage in EOL discussions. In others, the family wanted the diagnostic and prognostic information withheld from the patients due to a belief that (the patient) knowing the truth about the illness would cause the patient to lose hope. Sometimes, the patients recused themselves from decision making, especially common in women in certain cultures. Instead, they relegated decision-making to their family who may or may not use the principles of substituted judgment or, worse still, may act to promote their own personal interests over those of the patient's. This was particularly challenging where large extended families were involved, the hierarchy and process of decision-making was unclear or group decision-making was preferred. These situations typically triggered multiple time-intensive family meetings about EOL issues, a source of great stress for doctors. Faced with these complex situations, doctors who may not have the time and/or training to engage patients and families in a constructive manner may procrastinate or even avoid vital EOL conversations with their seriously ill patients.

### Barrier 5: Patient/family's limited health literacy

National data[30] show that limited health literacy disproportionately affects ethnic minorities, especially the poor, the uninsured and older adults. Research also shows that patients forget 40–80%[31] of medical information provided to them and half of what they do remember is incorrect[32]. Patients with limited health literacy may lack the ability to comprehend complex medical concepts like resuscitation preferences. Alternatively, they may have unrealistic expectations that medical technology is capable of curing any type of terminal illness or at least is effective in keeping the patient alive indefinitely with technological support.

Furthermore, it is very important to remember that even commonly used words can mean very different things to different people. For example, oncologists often use the word ***“cure”*** to indicate five years of cancer-free survival. However, to many cancer patients and families, the word ***“cure”*** means eradication of cancer and restoration of normal health. Another example is the English word 'hospice" which sounds like "***hospiscio***" in Spanish, meaning poorhouse. It is possible that when a doctor raises the option of referral to hospice care, an impoverished Spanish-speaking patient may misunderstand that the doctor is withholding expensive interventions and referring them to the poorhouse and thus be deeply offended. Such perceptions, whether merited or not, can create a deep mistrust in patients who may not vocalize these concerns but may resist end-of-life conversations with their doctors. The health literacy level of each patient needs to be assessed and clearly documented in the electronic medical records. Doctors need to be trained to avoid medical jargon when communicating with patients and use techniques like “teach back” [33] to assess for the patient’s understanding of information imparted.

### Barrier 6: Patient/family’s mistrust of doctors and the health care system

Because of historical events that have occurred between the medical establishment and certain ethnic communities, patients may not believe that doctors have their best interests at heart [34–36] and may fear abandonment or being subjected to poor quality care by self-interested medical professionals. Patient’s family may also believe that high-intensity interventions are being withheld or withdrawn from the seriously ill patient because of racism. Thus, any notions of withholding high-intensity medical interventions and instituting hospice care may be misinterpreted as deliberate provision of sub-standard care, leading the families to be deeply suspicious of EOL discussions. In situations where there is conflict about EOL decision-making, doctors (fearing litigation, family mistrust) automatically provide high-intensity interventions that may be ineffective and burdensome to terminally ill patients. Honest and open communication in simple language and shared decision making[37–38] will help foster trusting therapeutic alliances and thereby better quality EOL decision making. Additionally, public engagement campaigns are needed to educate Americans that high-intensity treatments on terminally ill patients do not necessarily result in prolonging life with quality but may only prolong the dying process while increasing the patient’s suffering.

To our knowledge, ours is the first and largest study of its kind using an innovative mixed methods approach to characterizing doctor-reported barriers to effective EOL conversations with diverse Americans. We were able to qualitatively identify the 6 main themes using a development cohort, empirically validate these themes by analyses of 50 randomly identified transcripts from the validation cohort and then finally use the results of the qualitative analysis to quantitatively analyze the validation cohort data. We believe that through this novel approach, we have demonstrated a strategy to employ rigorous quantitative research methods to assess the magnitude and frequency of constructs and rigorous qualitative research to explore the meaning and understanding of the constructs[39]. Furthermore we have merged quantitative and qualitative data to develop a more complete understanding of a complex and important problem[39]—in this case the barriers doctors face in conducting effective EOL conversations.

Our study is limited by the fact that the study participants were doctors from two hospitals in one geographic area. However, our institutions recruit and employ doctors from throughout the US and the ethnic and gender background of our doctors is reflective of the recent national trends of increasing women and diverse doctors reported by the Accreditation Council for Graduate Medical Education in 2013 (White 65.1%, Asian 21.2%, Black 6.3% and Hispanic American 6.3%). In fact, our diversity in doctors and the patients we serve was the main reason that made this study possible. Also, our study, is in part, a qualitative study and thus methodologically challenging to conduct nationally. However, it is to be noted that we have followed the COREQ[40] guidelines in conducting our study and in reporting our qualitative findings.

## Conclusion

Most doctors report that conducting effective EOL conversations with seriously ill patients is very challenging, especially with ethnic minority patients and their families. We have empirically identified the six primary barriers doctors report in conducting effective EOL conversations and demonstrated that the doctors’ age, ethnicity and sub-specialty influences their perceptions of these barriers. As the US is becoming increasingly diverse and as ethnic patients are more likely to consume ineffective and burdensome high-intensity treatments at the EOL, there is an urgent need to train doctors in conducting culturally effective EOL conversations early in the trajectory of any chronic and serious illness in order to facilitate dignity[41–42] at the EOL for diverse Americans.
